# Accumulation of chlorinated paraffins in adipocytes is determined by cellular lipid content and chlorination level

**DOI:** 10.1007/s00204-024-03956-3

**Published:** 2025-01-10

**Authors:** Nikola Vrzáčková, Jakub Tomáško, Petr Svoboda, Vojtěch Škop, Magdalena Melčová, Jana Dudová, Jaroslav Zelenka, Jana Pulkrabová, Tomáš Ruml

**Affiliations:** 1 Department of Biochemistry and Microbiology, University of Chemistry and Technology, Technická 3, 16000 Prague, Czech Republic; 2 Department of Food Analysis and Nutrition, University of Chemistry and Technology, Technická 3, 16000 Prague, Czech Republic; 3 Center for Experimental Medicine, Institute for Clinical and Experimental Medicine, Vídeňská 1958, 14021 Prague, Czech Republic

**Keywords:** Chlorinated paraffins, Persistent organic pollutants, 3T3-L1 cells, Adipocytes

## Abstract

Chlorinated paraffins (CPs) are environmental pollutants extensively used in industries. While the use of short-chain chlorinated paraffins (SCCPs) has been restricted since 2017, the use of medium-chain chlorinated paraffins (MCCPs) has risen as their replacement. Due to lipophilic character, it can be expected that CPs enter the cells; however, the in vitro accumulation potential of CPs remains poorly understood. In this study, we aimed to explore the ability of SCCPs and MCCPs to accumulate in fat cells. We utilized an in vitro model of mouse 3T3-L1 preadipocytes and adipocytes. Using gas chromatography coupled with high-resolution mass spectrometry operated in negative chemical ionization mode, we determined the intracellular amounts of CPs. These compounds accumulated at rates of 8.5 ± 0.1 µg/g_cells_/h for SCCPs and 7.8 ± 0.3 µg/g_cells_/h for MCCPs when an initial concentration of 120 ng/ml was present in the medium. This rate increased approximately tenfold when the concentration of CPs was raised to 1200 ng/ml. CPs content in adipocytes steadily increased over 5 days, whereas preadipocytes accumulated 15–20 times less CPs. This highlights the importance of cellular lipid content, which was about 12 times higher in adipocytes. Furthermore, we found that the level of chlorine content in the CPs molecules significantly influenced their accumulation. Our results demonstrate that MCCPs exhibit a similar accumulation potential to SCCPs, with lipid content playing a crucial role. As with SCCPs, restrictions on the use of MCCPs in industry should be considered to mitigate their environmental and health impacts.

**Supplementary Information:**

The online version contains supplementary material available at 10.1007/s00204-024-03956-3.

## Introduction

Chlorinated paraffins (CPs), successors of the infamous polychlorinated biphenyls (PCBs), are emerging as significant environmental pollutants. These compounds are polychlorinated *n*-alkanes synthesized through the radical chlorination of straight-chained alkane fractions. CPs are commonly classified by the length of their carbon chains into three groups: short-chain (C_10–13_; SCCPs), medium-chain (C_14–17_; MCCPs), and long-chain CPs (C_18–30_; LCCPs) (Krätschmer et al. 2018). Dominant components of CPs are polychlorinated *n*-alkanes (PCA) and PCA are also the most commonly analyzed compounds in CPs technical mixtures (Fernandes et al. 2023). Nonetheless, CPs (containing not only PCA, but other compounds and byproducts of synthesis) are used in industry applications and are subsequently present in the environment and biota. Therefore, the analytes would be referred as CPs in this work as the impurities may influence the viability of cells.

SCCPs have found extensive use in various industrial applications, including metalworking fluids, paints, sealants, lubricant additives, flame retardants, and plasticizers. Their popularity originated from their desirable physical and chemical properties, such as thermal stability, variable viscosity, flame resistance, and low vapor pressure (Glüge et al. 2016). However, these same properties contribute to their environmental persistence. Studies have documented the widespread occurrence of CPs in the environment, their long-range transport, bioaccumulation in the food chain, and toxicity to aquatic organisms (Bezchlebova et al. 2007; Tomy et al. 2000). Consequently, SCCPs were included into the Stockholm Convention in 2017 as persistent organic pollutants and subsequently banned in the European Union and the United States (UNEP/POPS/COP8/INF/19, 2017).

The ban on SCCPs led to an increased use and production of MCCPs as substitutes due to their similar physical and chemical properties, which resulted in rising MCCPs pollution. Notably, the potential toxicity of MCCPs and LCCPs has been largely overlooked (van Mourik et al. 2016). Despite the scarcity of data, existing research suggests that MCCPs and LCCPs may exhibit biological effects akin to those of SCCPs (Darnerud and Bergman 2022). In a seminal study by Bucher et al. (1987), chronic high-dose administration of both SCCPs and LCCPs showed serious pro-inflammatory and carcinogenic effects in rats and mice though with different tissue targeting. As a result, there is a growing call to add MCCPs to the Stockholm Convention and the MCCPs are currently being assessed to be included in the Convention (UNEP, 2022). However, there remains a significant lack of information about the accumulation and toxicological properties of MCCPs compared to SCCPs, particularly concerning the impact of chain length and chlorination level on their accumulation.

Here, we used differentiated 3T3-L1 adipocytes as a model for adipose tissue to investigate the accumulation properties of both MCCPs and SCCPs at doses observed in population studies (Tomasko et al. 2021). By employing a mixture of different CPs congeners, we aimed to elucidate the effects of varying carbon chain lengths and chlorine content on their accumulation potential. This research is essential for understanding the environmental and health risks posed by these widespread pollutants.

## Materials and methods

### Chemicals and materials

Mixtures of SCCPs (C_10–13_ 63.0% Cl *w/w*, 100 µg/ml cyclohexane), MCCPs (C_14–17_ 52.0% Cl and 57.0% Cl *w/w*, both 100 µg/ml cyclohexane) and PCB 166 (10 µg/ml isooctane; used as the internal standard in the analysis of CPs) were obtained from LGC Standards (Teddington, UK). The CPs mixtures were chosen for the experiments as they more closely represent the CPs contaminating the environment and biota than PCA standards. Dichloromethane (≥ 99.8%), isooctane (≥ 99.8%), diethyl ether (≥ 99.7%), florisil (particle size 0.15–0.25 mm; baked at 600 °C for 4 h before use), and sulfuric acid (≥ 98.0%) were all purchased from Merck (Darmstadt, Germany). Acetonitrile (≥ 99.9%) and *n*-hexane (≥ 97.0%) were obtained from Honeywell Riedel-de Haën™ (Charlotte, USA). Sodium sulfate (≥ 99.0%; baked at 600 °C for 6 h before its use) was obtained from Penta s.r.o. (Prague, Czech Republic). Deionized water was prepared using a Milli-Q water purification system (Merck, Darmstadt, Germany). Technical gasses (nitrogen 5.0 and 4.0, helium 6.0, and methane 5.5) were supplied by SIAD (Prague, Czech Republic).

Mouse 3T3-L1 embryonic fibroblasts were purchased from the American Type Culture Collection (USA, Manassas, VA). The rest of the material and chemicals was from Gibco (USA, Carlsbad, CA) unless otherwise indicated.

### Cell culture and differentiation to adipocytes

Mouse 3T3-L1 embryonic fibroblasts were routinely cultured in Dulbecco’s modified Eagle’s medium (DMEM; cat. no. 21969035) containing 4.5 g/l d-glucose and 1 mmol/l sodium pyruvate and supplemented with 4 mmol/l l-glutamine, 10% fetal bovine serum (FBS) and the mixture of 100 U/ml penicillin and 100 µg/ml streptomycin sulfate in the humidified atmosphere with 5% CO_2_ at 37 °C.

Non-differentiated 3T3-L1 cells are referred to as preadipocytes. The 3T3-L1 cells after differentiation protocol are referred to as adipocytes.

**Fig. 1:**
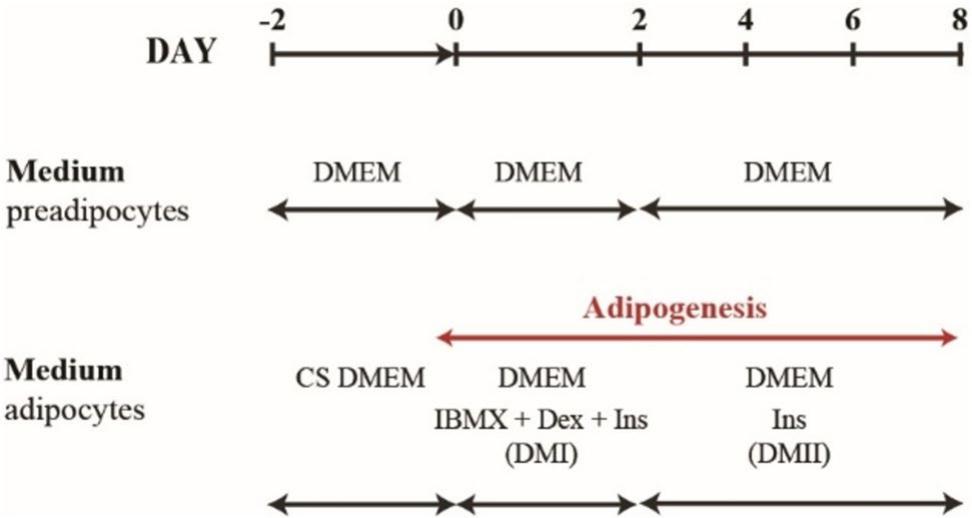
Scheme of differentiation—CS (calf serum), Dex (dexamethasone), DMEM (Dulbecco’s modified Eagle’s medium), DMI (differentiation medium I), DMII (differentiation medium II), FBS (fetal bovine serum), IBMX (3-isobutyl-1-methylxanthine), Ins (insulin)

The cells were differentiated into mature adipocytes as described previously (Skop et al. 2014; Vacurova et al. 2022). Briefly, the cells were seeded in 6-well plates at 1·10^5^ cells/well density and incubated for 48 h to reach an absolute confluence. Then, the routine DMEM with 10% calf serum (CS) instead of FBS was applied for another 48 h. Differentiation of 3T3-L1 into adipocytes was started (day 0) by adding differentiation medium containing routine DMEM with a cocktail of differentiation inducers 0.5 mmol/l 3-isobutyl-1-methylxanthine (Merck, Darmstadt, Germany), 1 μmol/l dexamethasone (Merck), 1.7 μmol/l insulin (Merck, Darmstadt, Germany), and 25 μmol/l HEPES for another 48 h (day 0–2). Then, the differentiation medium was switched for the second differentiation medium containing DMEM with 1.7 μmol/l insulin and 25 μmol/l HEPES. This medium was changed every 48 h for 6 days (2–8). The differentiation and experiment time schedule is in Fig. 1.

### Preparation of medium with CPs

The mixture of SCCPs (63.0% Cl content), MCCPs (52.0% Cl content) and MCCPs 57.0% Cl content) was prepared in a ratio of 2:1:1 (*w/w/w*). Cyclohexane was evaporated by a gentle stream of nitrogen, and the mixture was then dissolved in methanol and added to the culture medium, reaching constant 0.1% methanol in the medium. Each experiment listed below was performed in routine DMEM without phenol red and with 20% FBS. After the experiment cells and media were harvested by pipetting out the medium, washing cells with Dulbecco’s Phosphate-Buffered Saline (Biosera, Nuaille, France; PBS), and scraping off the cells. Harvested cells and media were stored at − 20 °C.

### Measurement of viability

Mouse 3T3-L1 cells were seeded in 96-well plates at the concentration of 5·10^3^ cells/well. Experiment was performed on both preadipocytes and mature adipocytes. At day 8, mixture of CPs was added in concentrations 0–20,000 ng/ml. Medium with/without CPs was changed every 24 h and viability was measured on day 1, 3 and 7 after the first addition of CPs.

**Fig. 2:**
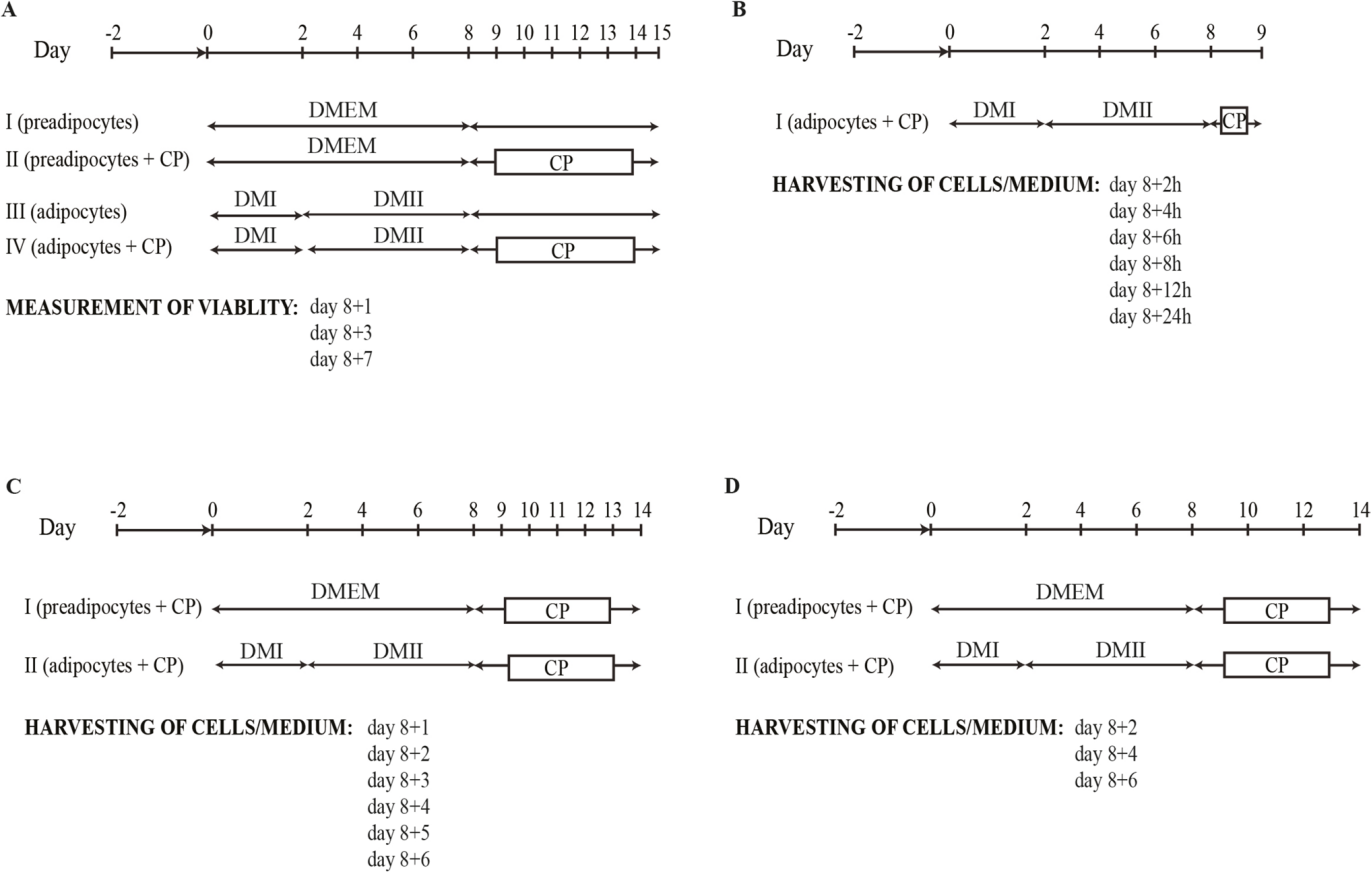
Schemes of experiments—CPs (chlorinated paraffins), DMI (differentiation medium I), DMII (differentiation medium II), DMEM (Dulbecco’s modified Eagle’s medium)

Cell viability was measured as described previously (Trnovska et al. 2021) using the Cell Proliferation Reagent WST-1 (Roche Diagnostics GmbH, Mannheim, Germany). Absorbance was measured at 450 nm with reference absorbance at 650 nm. The experiment time schedule is in Fig. 2A.

### Measurement of differences in rate accumulation of CPs in time

Mouse 3T3-L1 cells were seeded in 6-well plates at 1·10^5^ cells/well. Cells were differentiated to adipocytes. At day 8, mixture of CPs was added in two concentrations—120 and 1200 ng/ml. After this one-time addition cells and media were harvested at 2, 4, 6, 8, 12 and 24 h after the first addition of CPs. Also, experimental group without any addition of CPs was harvested. The experiment time schedule is in Fig. 2 B.

### Measurement of cellular lipids content and chlorine levels/carbon chain length on CPs accumulation

Mouse 3T3-L1 cells were seeded in 6-well plates at 1·10^5^ cells/well. Experiment was performed on preadipocytes as well as on the mature adipocytes. At day 8, mixture of CPs was added to the preadipocytes/adipocytes in two concentrations—50 and 500 ng/ml. Medium with CPs was changed every 12 h and cells and media were harvested for total of 6 days every 24 h after the first addition of CPs. The experiment time schedule is in Fig. 2C.

For the measurement of lipid content, both cells and media from separate experiment with the same time layout were used. Prior to the triacylglycerols measurement, cells were scraped to in PBS and then lysed by sonication for 15 s. Triacylglycerols (TAG) were quantified using an enzymatic kit (Erba-Lachema). A total volume of 10 μl of cell lysate or medium was used for each measurement on 96-well plate. Subsequently, 200 μl of reaction mix from the kit was added to the well, and the cells were incubated at 37 °C for 30 min. Absorbance was then measured at 540 nm wavelength. For quantification, a TAG stand was used. Using this method, CPs level could be adjusted for cellular lipid content.

### Measurement of distribution pattern of CPs

Mouse 3T3-L1 cells were seeded in 6-well plates at 1·10^5^ cells/well. Experiment was performed on preadipocytes as well as on the mature adipocytes that were differentiated using described differentiation protocol. At day 8, a mixture of CPs was added to the preadipocytes/adipocytes in two concentrations—120 and 1200 ng/ml. Medium with CPs was changed every 48 h and cells and media were harvested at day 2, 4 and 6 after the first addition of CPs. The experiment time schedule is in Fig. 2D.

### Analysis of adsorption/evaporation rate of CPs in wells

CPs were added to the medium in the 6-well plates without any cells seeded. The plates were then incubated under the same condition as in previous experiments—humidified atmosphere with 5% CO_2_ at 37 °C for 48 h. After this period, volume of medium was measured and CPs in this volume were quantified.

### Sample preparation for the analysis of CPs

Before extracting CPs from the cells, the harvested cultures were lyophilized using Freeze Dryer ALPHA 2–4 LDplus (Martin Christ, Osterode am Hartz, Germany). Then, PCB 166 in isooctane (0.25 ml; 5 ng/ml) was added, and the samples were thoroughly vortexed. To release all CPs from the cells and to hydrolyze the lipids, 50 µl of concentrated sulfuric acid was added, and the samples were vortexed again for 30 s. After centrifugation (3186 × *g* for 2 min), the upper organic layer was transferred into a vial and further analyzed by gas chromatography coupled with high-resolution mass spectrometry operated in negative chemical ionization mode (GC-NCI-HRMS).

Samples of culture medium (1 ml of medium collected after cell incubation with CPs was pipetted into a 15 ml centrifuge tube) were mixed with 1 ml of acetonitrile to precipitate the proteins, and the CPs were then extracted with 3 ml *n*-hexane:diethyl ether (9:1, *v/v*), shaken for 2 min and centrifuged at 3186 × g for 2 min. The upper organic layer (2.4 ml) was transferred into a 5 ml vial, and the extraction was repeated once more with a 1.5 ml *n*-hexane:diethyl ether (9:1, *v/v*) addition to the original tube. After the second extraction, 1.8 ml of the organic phase was pooled with the first aliquot in the vial. It was then concentrated to 0.2–0.3 ml under a gentle stream of nitrogen. The residual water and other possible coextracts were removed from the combined extract by solid-phase extraction on a multilayer column (from bottom to the top filled with glass wool, 0.4 g florisil, and 1 g anhydrous sodium sulfate). The CPs were eluted by *n*-hexane:dichloromethane (3:1, *v/v*), the obtained eluate was evaporated by a vacuum rotary evaporator, and the residual solvent was dried under a gentle stream of nitrogen. The sample was then dissolved in a 0.25 ml syringe standard (PCB 166, 5 ng/ml of isooctane), and the CPs were analyzed by GC-NCI-HRMS.

### GC–NCI–HRMS analysis

The analysis of SCCPs and MCCPs was carried out on a gas chromatograph Agilent 7890B coupled with Agilent 7200B quadrupole-time of flight mass spectrometer (GC/Q-TOF system; both Agilent Technologies, Santa Clara, USA) operated in a negative chemical ionization mode with resolution of < 10,000 (FWHM at 185 m/*z*) [11].

The monitored masses are described elsewhere (Tomasko et al. 2023). The standards of CPs are described by a total concentration of all components, with no further information about the concentrations of individual compounds. The content of congener groups (described by a molecular formula) can be described by internal normalization (ratio of congener group area divided by a total area of either total CPs or only SCCPs or MCCPs). In this study, CPs profiles were made for SCCPs and MCCPs separately, as the response factors for SCCPs and MCCPs differs strongly on the used GC–MS instrumentation. On the one hand, the concentrations should not be derived from relative abundances without proper calibration by standards and also the comparison of profiles obtained in different laboratories might be hardly comparable, due to the influence of instrumentation and measurement conditions on CPs signals. On the other hand, the changes in CPs profiles obtained under the same conditions might hint a change in CPs composition, which would be too slight to be seen on a change of total concentrations.

The quantification was done by external calibration curve method. The calibration standard was prepared from a mixture in methanol used for spiking of cell media and it was diluted to levels 5, 10, 20, 50, 200, 500, and 1000 ng/ml isooctane. The total responses of either SCCPs or MCCPs were used. For the quantification of CPs in adipocytes and preadipocytes, matrix calibration points were prepared (by extracting blank cells) because of strong matrix effects. CPs in media were quantified by solvent calibration points, which was considered sufficient according to the validation parameters (see QA/QC section). The CPs are usually quantified by special procedures because of the nature of CPs composition. In this case, we were quantifying mixtures of known origin and composition, which have not changed significantly during the experiments (in the point of view of instrument response).

### Determination of CPs and their possible biotransformation products

The data obtained by GC–NCI–HRMS were processed with MassHunter Quantitative Analysis software (B.10.1.733.0; Agilent Technologies, Santa Clara, USA). The monitored exact masses have been described elsewhere (Tomasko et al. 2021). Potential biotransformation products were screened in the measured data of preadipocytes and adipocytes after 7 days. The possible transformation products were selected according to He et al. (2021), i.e., CPs with shorter carbon chains, alcohols, ketones, and carboxylic acids. The above study used a direct MS (without chromatographic separation) to monitor the [M-Cl]^−^ ions. The exact masses were therefore recalculated to [M–Cl]^−^ formations limited to 600 m/z.

### QA/QC in the analysis of CPs

Before starting the experiments, the used materials were tested for the presence of CPs (DMSO, cells, fetal bovine serum, medium), and no SCCPs and MCCPs were detectable.

During the experiments, procedural blanks were analyzed in each batch—apart from the analysis of the control samples (blank medium and cells without exposure to CPs). The procedural blanks for the analysis of cells were all without any CPs contamination. The procedural blanks for the analysis of medium contained small amounts of CPs, just below the limits of quantification. The areas of detected CPs in blanks were subtracted from the samples.

The recovery and repeatability of the method for the analysis of CPs in media were evaluated by analyzing spiked samples at two different concentrations (10 and 100 ng/ml of media for SCCPs and 20 and 200 ng/ml media for MCCPs) in six parallel determinations (each level). The recoveries were 79% and 95% (SCCPs) and 99% and 119% (MCCPs). The repeatability (expressed as relative standard deviations—RSD) was 9% and 5% (SCCPs) and 9% and 7% (MCCPs).

### Statistical analysis

The data are expressed as the mean ± SD. Differences among variables were evaluated by one-way ANOVA. Statistical analysis was performed using Graph Prism 9.0.0 (GraphPad, CA, USA). The differences between CPs profiles were evaluated by paired t-tests using MS Excel (Microsoft, USA). Differences were considered statistically significant at *p* < 0.05 in both cases.

Heat maps were created using the ggplot function [H. Wickham. ggplot2], and linear regression analysis was performed using the lm function, both in R [R Core Team (2023)]. First, linear regression between time (days) and the relative quantity changes of each congener was performed to assess whether some congeners accumulate more than others. Slopes from these regressions were recorded and used in the next step, where these slopes of relative quantity changes were modeled using linear regression with the formula: slope (relative quantity change over time) ~ length (number of carbon atoms) + chlorine content (number of chlorine atoms).

## Results

### SCCPs and MCCPs accumulate in adipocytes at similar rates

**Fig. 3:**
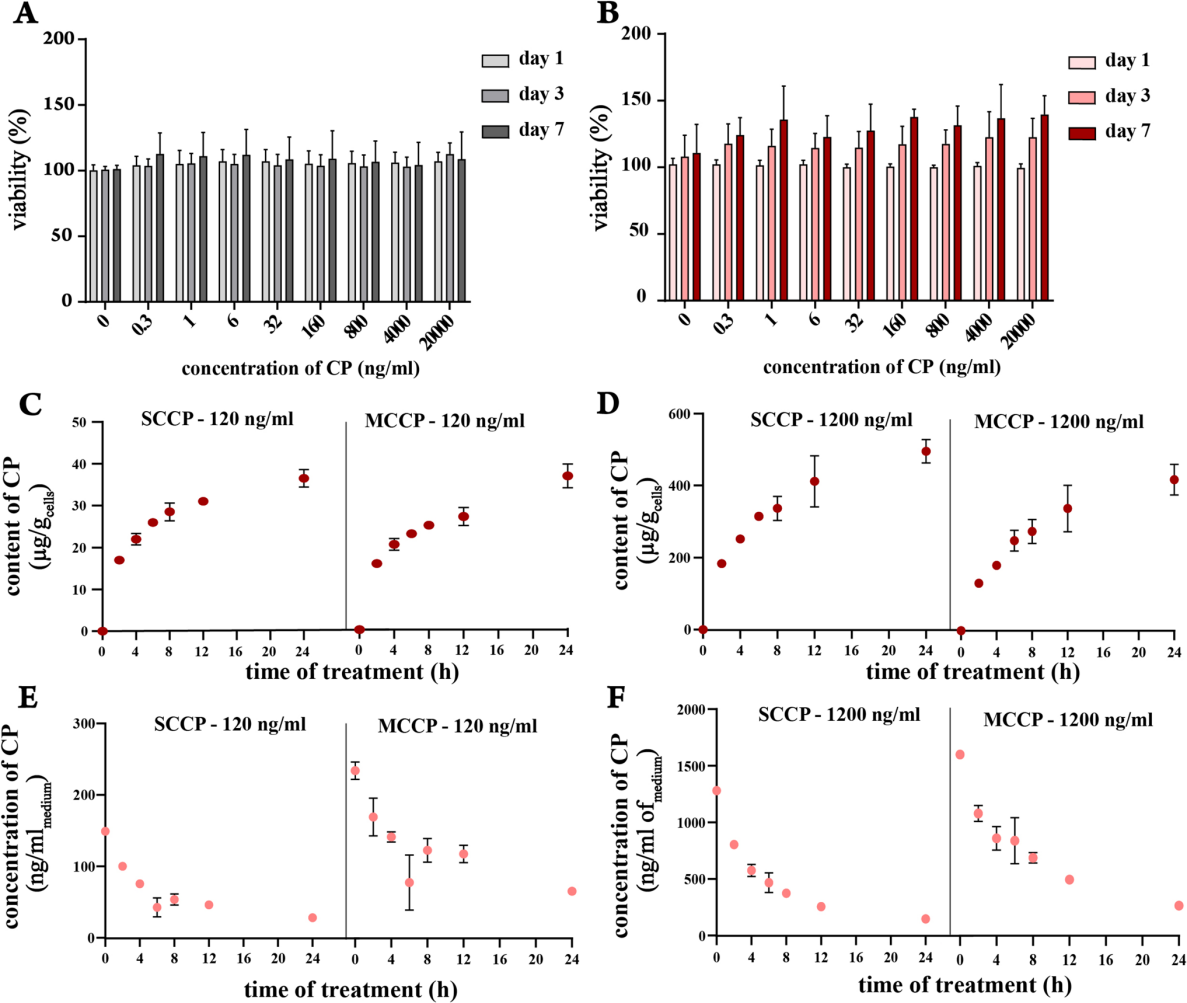
SCCPs and MCCPs accumulation in 3T3-L1 adipocytes. **A**, **B**: 3T3-L1 preadipocytes (**A**) and adipocytes (**B**) were treated with different concentrations of CPs mixtures. CPs were added to the culture media every 24 h. Viability was measured on day 1 after the initial CPs addition and again on days 3 and 7 using the WST-1 assay. **C**–**F**: CPs were added to the culture media of 3T3-L1 adipocytes at two concentrations: 120 ng/ml (**C**, **E**) and 1200 ng/ml (**D**, **F**). Cells and media were collected at 2, 4, 6, 8, 12, and 24 h post-CPs addition. Panels C and D show the cellular content of SCCPs and MCCPs, respectively, while panels E and F show the concentrations of SCCPs and MCCPs in the media. Data are presented as mean ± SD, *n* = 3

We first ensured that the CPs concentrations used in this study do not cause acute toxicity or reduce cell viability. Concentrations up to 20,000 ng/ml did not affect the viability of 3T3-L1 preadipocytes or adipocytes over 1, 3, and 7 days of treatment (Fig. 3A and B), confirming the suitability of this concentration range for accumulation studies.

The use of short-chain chlorinated paraffins (SCCPs) has been restricted due to their toxicological concerns and environmental persistence (UNEP/POPS/COP8/INF/19, 2017), while medium-chain chlorinated paraffins (MCCPs) currently do not face such restrictions. To explore the differences in accumulation potential between these two groups, we studied their accumulation in 3T3-L1 adipocytes. A single dose of 120 ng/ml SCCPs added to the cultivation media led to a steady increase in their cellular content over 24 h. The highest accumulation rate of SCCPs, reaching 8.5 ± 0.1 µg/g_cells_/h, was observed at the beginning of the treatment. This rate and the amount of SCCPs accumulated over 24 h were approximately 10 times higher when the initial dose was 1200 ng/ml, indicating a proportional relationship between concentration and accumulation rate (Fig. 3C). MCCPs accumulated at a similar rate like SCCPs, with accumulation rates of 7.8 ± 0.3 µg/g_cells_/h (initial dose: 120 ng/ml) or 64.3 ± 1.3 µg/g_cells_/h (initial dose: 1200 ng/ml) observed during the first two hours of treatment (Fig. 3D), demonstrating similar accumulation abilities of these two CPs groups in adipocytes. Concurrently, the concentration of CPs in the cultivation media substantially decreased as their cellular content increased. Regardless of the initial concentration, only 15–30% of the original amount of both SCCPs and MCCPs remained in the media after 24 h (Fig. 3E, F). When medium containing 1200 ng/ml of CPs was placed in a culture well without cells, a reduction of ~ 10 ng/ml/h was noted. The reduction could be both evaporation-induced and sorption on well walls. No reduction occurred with a starting concentration of 120 ng/ml (Suppl Fig. 1). These measured rates of reduction suggest that cellular accumulation accounted for most (> 90%) of the decrease in CPs in the culture media. In summary, SCCPs and MCCPs rapidly accumulate in adipocytes.

### Cellular lipid content determines CPs accumulation

**Fig. 4:**
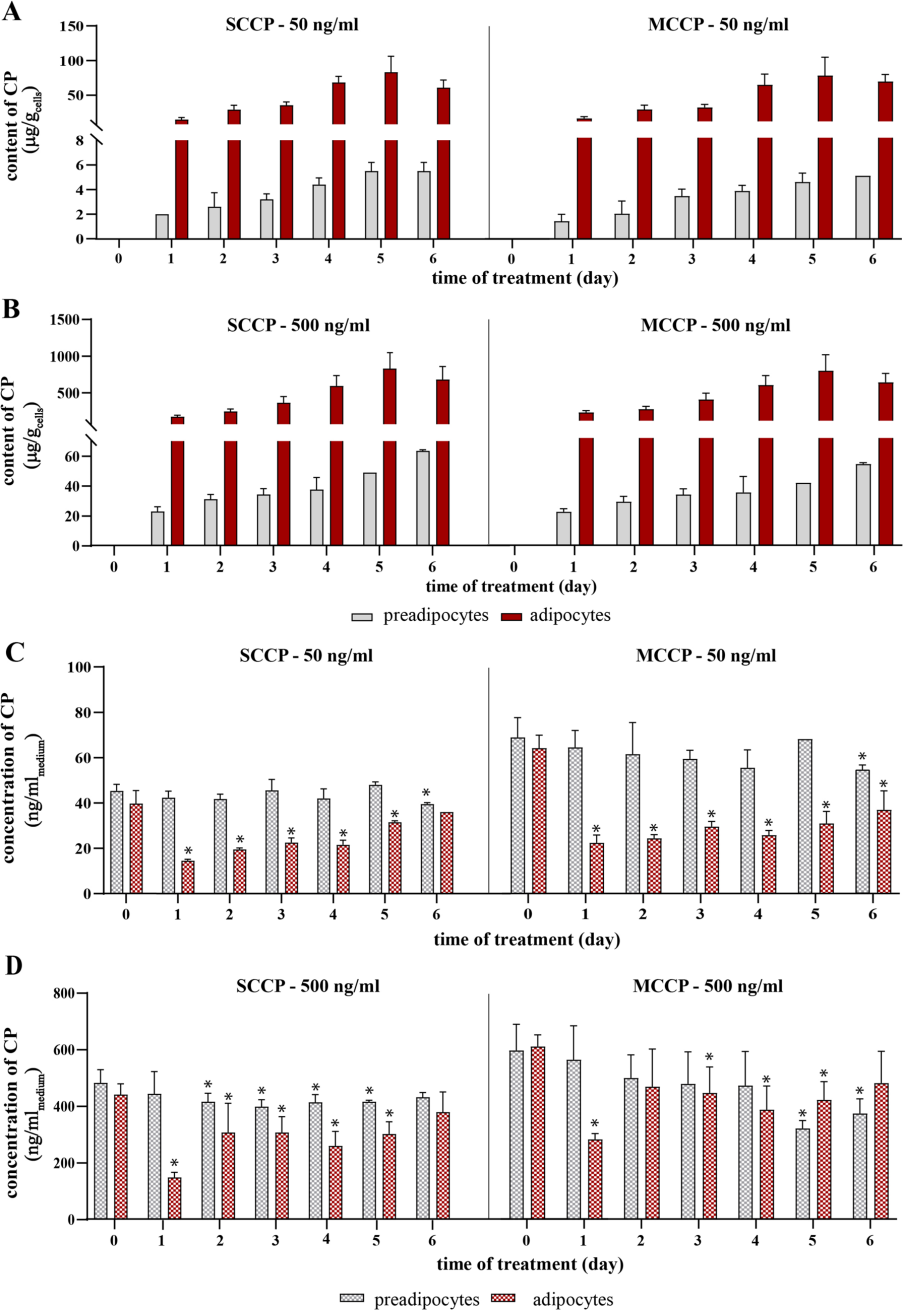
Long-term accumulation of SCCPs and MCCPs in adipocytes and preadipocytes. CPs were added to the culture media of 3T3-L1 preadipocytes and adipocytes at two concentrations: 50 ng/ml (**A**, **C**) and 500 ng/ml (**B**, **D**). Media were exchanged every 12 h. Cells and media were collected after 1, 2, 3, 4, 5, or 6 days from the first CPs addition. Panels A and B show the cellular content of SCCPs and MCCPs in preadipocytes and adipocytes, respectively. Panels C and D show the concentrations of SCCPs and MCCPs in the media. Data are presented as mean ± SD, *n* = 5, *p value < 0.05 compared to day 0 separately for preadipocytes and adipocytes

To explore how cellular lipids affect CPs accumulation, we treated both 3T3-L1 preadipocytes and 3T3-L1 adipocytes (which had about 12 times more lipids compare to preadipocytes; Suppl. Figure 2F) with 50 and 500 ng/ml of SCCPs and MCCPs for 6 days. We replaced the CPs-containing media every 12 h to maintain sufficient concentration and remove possible byproducts. With 500 ng/ml of SCCPs or MCCPs in the media, the CPs content in adipocytes steadily increased over 5 days, peaking at concentrations of 829 ± 207 µg/g_cells_ for SCCPs and 730 ± 192 µg/g_cells_ for MCCPs. Consistent with our earlier findings, the maximum amounts of CPs accumulated in the cells were proportional to the CPs concentrations in the media and were similar between SCCPs and MCCPs. In contrast, preadipocytes accumulated 15–20 times less CPs compared to adipocytes, and their accumulation did not reach a maximum (Fig. 4A, B). There was also no detectable decrease in SCCPs or MCCPs concentration in the preadipocyte media (Fig. 4C, D). When the CPs level was adjusted for cellular lipid content, preadipocytes accumulated 102 ± 0.1 μg SCCPs/g_lipids_ and 75 ± 0.1 μg MCCPs/g_lipids_ over 5 days with a CPs media concentration of 500 ng/ml. This accumulation is only slightly less than that observed in adipocytes, which accumulated 144 ± 36 μg SCCPs/g_lipids_ and 127 ± 33 μg MCCPs/g_lipids_, underscoring the critical role of cellular lipids in CPs accumulation.

**Fig. 5:**
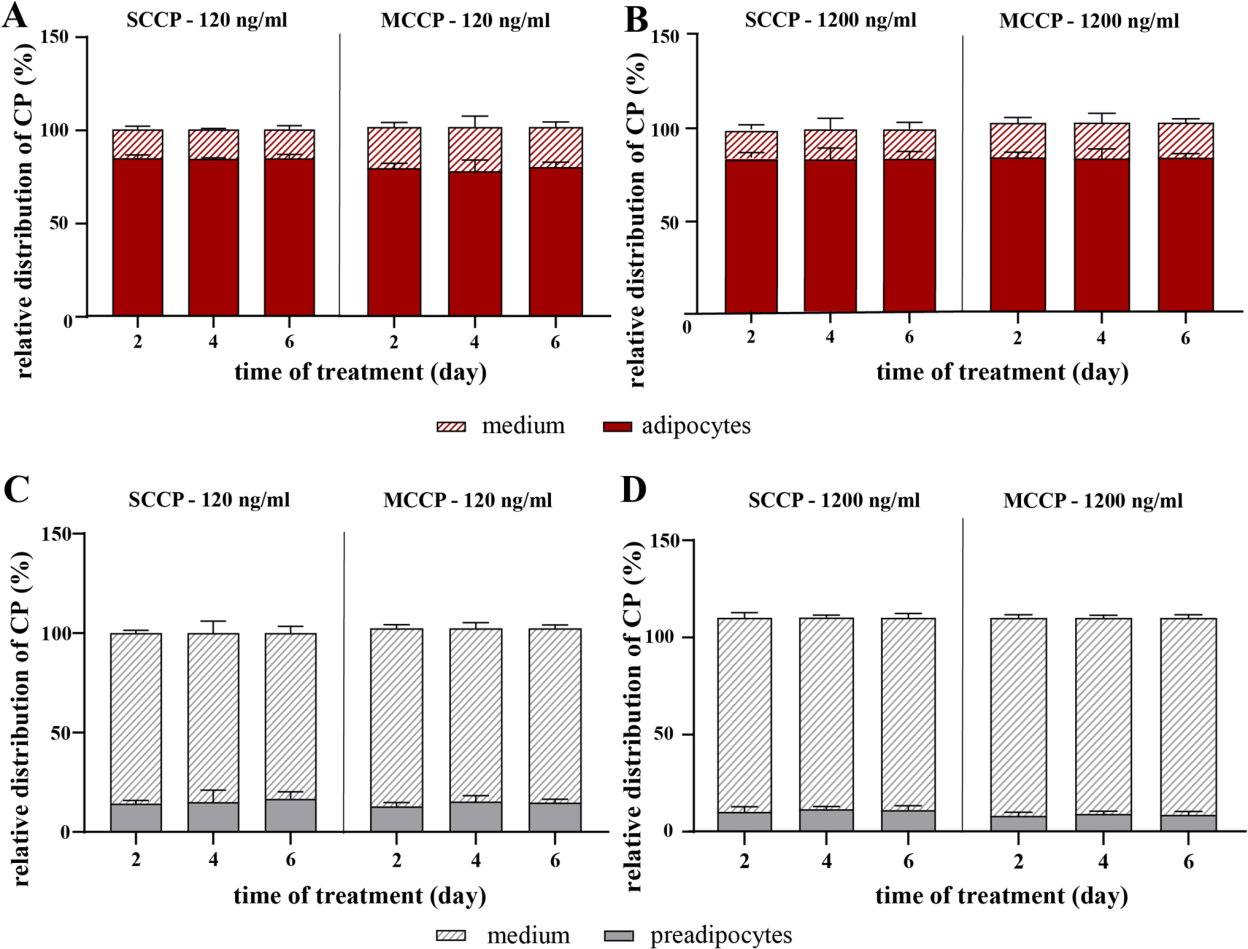
Distribution of SCCPs and MCCPs between media and cells. CPs were added to the culture media of 3T3-L1 adipocytes (**A**, **B**) or preadipocytes (**C**, **D**) at two concentrations: 120 ng/ml (**A**, **C**) and 1200 ng/ml (**B**, **D**). Media were exchanged every 48 h. Cells and media were collected after 2, 4, or 6 days from the first CPs addition. The quantity of CPs in cells and media was determined using GC-NCI-HRMS and expressed as the relative amount present in media and cells. Data are presented as mean ± SD, *n* = 3

To quantify the contribution of intracellular lipids to CPs accumulation, we assessed the distribution of CPs between the media and cells in 3T3-L1 preadipocytes and adipocytes. Cells were treated with either 120 or 1200 ng/ml of SCCPs or MCCPs, and the media were replaced every 48 h. The CPs content in both cells and media was measured at each 48 h interval. When the initial concentration of SCCPs or MCCPs was 120 ng/ml, 84 ± 0.5% of SCCPs and 77 ± 5.0% of MCCPs were found inside adipocytes after two days. Prolonged exposure to CPs did not substantially alter these distribution patterns (Fig. 5A). A similar ratio (80–90%) of SCCPs and MCCPs content between cells and media was observed when the CPs concentration was increased to 1200 ng/ml (Fig. 5B), suggesting that at these ratios, intracellular and extracellular CPs contents are nearly in equilibrium. Each cultivation well contained approximately 16 mg of adipocytes and 2 g of media. At equilibrium, the cellular content (in µg/g_cells_) of SCCPs and MCCPs was ~ 600 times higher than their concentration in the media. In contrast, in 3T3-L1 preadipocytes, only about 10% of SCCPs and MCCPs were found intracellularly (Fig. 5C, D). Therefore, in these cells, at equilibrium, the cellular content (in µg/g_cells_) of SCCPs and MCCPs is only about 35 times higher than their concentration in the media. These findings indicate that cellular lipids are essential for SCCPs and MCCPs accumulation and that the accumulated amount is proportional to the lipid content.

### Chlorination level, but not chain length, affects the accumulation ability of CPs

**Fig. 6:**
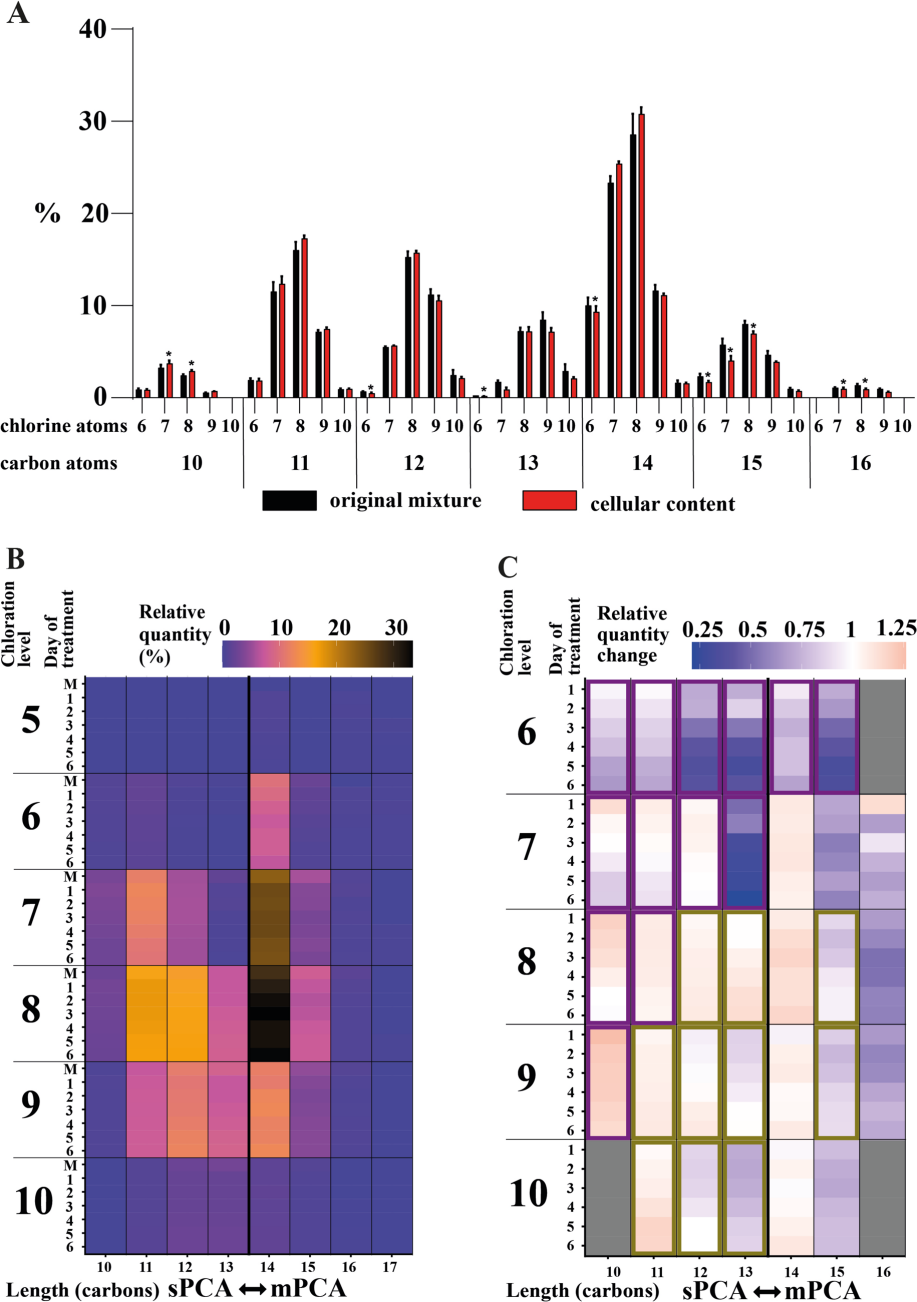
Effect of carbon chain length and chlorination level on PCA accumulation. CPs were added to the culture media of 3T3-L1 adipocytes at a concentration of 500 ng/ml and replaced every 24 h. The content of individual PCA congeners inside cells was determined daily over 6 days. To control for handling, the composition of the mixture (M) was determined by mixing it with the cell pellet and immediate analysis. **A**: Comparison of the relative quantity of individual SCCPs or MCCPs congeners at baseline and after one day of treatment. Data are presented as mean ± SD, *n* = 3–4, *p* value < 0.05 compared to the original mixture. **B**: Relative quantity of each SCCPs or MCCPs congener organized by the number of carbon and chlorine atoms at each day of the experiment. The heat map uses a color gradient to represent the relative percentual quantity of each SCCPs or MCCPs congener, with totals set to 100% for each group per day. **C**: To correct for different quantities of congeners in the mixture, the quantity of each congener in cells was expressed as a fold change compared to the original mixture. The heat map uses a color gradient to show the fold change in the quantity of each SCCPs or MCCPs congener in cells, relative to the original mixture (panel B, labeled M). Linear regression between this relative quantity fold change and the day of treatment was used to determine whether the quantity of each congener over time was significantly decreasing (encircled in purple) or increasing (encircled in olive). **B**, **C**: Data are presented as mean, *n* = 3–4

SCCPs and MCCPs have a similar ability to accumulate in adipocytes. However, both groups consist of numerous congeners and are typically used in mixtures. The accumulation properties of different congeners were investigated to determine whether some of the congeners are accumulated preferentially and what features contribute to this preference. 3T3-L1 adipocytes were treated with a 1:1 (*w/w*) mixture of SCCPs and MCCPs. The relative quantities of individual CPs congeners accumulated in the cells after 24 h were compared with their quantities in the original mixture (Fig. 6A). It was found that the most abundant congeners in cells contained 11, 12, and 14 carbon atoms and 7–9 chlorine atoms, representing 65% of the total detected CPs by weight. There were subtle changes in the relative quantities of individual congeners in cells compared to the original mixture, but no clear pattern of the effect of chain length or chlorination level was observed.

To better describe the effect of chain length and chlorination level on intracellular CPs accumulation, the cells were cultivated with the same CPs mixture for an additional 5 days. During this period, the cellular CPs composition was measured and the CP-containing media was replaced daily (Fig. 6B). Changes in CPs profiles were monitored, based on the relative abundances. Linear regression between days and the relative abundances was used to determine whether a congener’s relative abundances increased or decreased over time (Fig. 6). Interestingly, the relative abundances of congeners with fewer chlorine atoms (6 or 7) decreased (congeners with 5 chlorine atoms were not detected), while the relative abundances of more highly chlorinated congeners (with 8 and more chlorines) increased. Despite this effect being more pronounced in SCCPs, a similar pattern was observed in MCCPs. The slope of the relative abundances change was modeled using a linear model with chlorination level and chain length as inputs for SCCPs and MCCPs separately (Table 1). The coefficient of determination (*R*^2^) of this model was 0.78 for SCCPs and 0.69 for MCCPs, suggesting that in both groups, these two parameters determine most of a congener’s ability to accumulate in adipocytes. Interestingly, chain length was not a significant factor in either SCCPs or MCCPs. On the other hand, chlorination level had a highly significant effect in both CPs groups (*p* value < 0.01), suggesting that chlorine content, rather than chain length, determines the ability for deposition, with higher chlorine content leading to increased accumulation.

**Table 1:** Linear model output

CPs group	Parameter	Slope*	*p* value	*R*^2^
SCCPs	Length	0.0066 ± 0.0048	0.19	0.7789
	Cl content	0.0271 ± 0.0038	0.000002	
MCCPS	Length	− 0.0089 ± 0.0067	0.21	0.6919
	Cl content	0.0199 ± 0.0046	0.0011	

The relative quantity change (from Fig. 6C) was modeled using a linear regression model with two parameters: number of carbon atoms (Length) and number of chlorine atoms (Cl content)

*Units of slope is Relative quantity change/day/number of atoms of carbon (Length) or chlorine (Cl content)

Overall, the data suggest that while all CPs accumulate in adipocytes, there are differences in the accumulation abilities of individual congeners. The primary determinant of this ability is the chlorine content.

## Discussion

Chlorinated paraffins (CPs) are persistent environmental pollutants that pose potential health risks, making it essential to understand how they accumulate in biological systems. Our study shows that at non-toxic concentrations, both short-chain chlorinated paraffins (SCCPs) and medium-chain chlorinated paraffins (MCCPs) accumulate in adipocytes at similar rates. Higher doses lead to proportionally greater accumulation. The lipid content in cells is crucial for CPs accumulation, as adipocytes accumulated significantly more CPs than preadipocytes and this accumulation was proportional to lipid content. Notably, our findings indicate that the level of chlorination, rather than chain length, determines the accumulation ability of individual congeners. Congeners with higher chlorination levels showed increased accumulation in adipocytes. Overall, these results highlight the importance of cellular lipids and chlorination levels in the accumulation of CPs and show no significant differences in the accumulation capabilities between SCCPs and MCCPs.

EFSA has evaluated the risks of different CPs variants in food and feed. SCCPs and MCCPs primarily target the liver, kidney, and thyroid in toxicity studies. In animal experiments, effects, such as decreased pup survival and increased kidney weights, were observed. The liver was the primary target organ for LCCPs, although human-relevant reference points for LCCPs were not identified due to data limitations. Dietary exposure to CPs through food, particularly fish, was preliminarily assessed. While no health concerns were suggested in limited scenarios, EFSA emphasized that dietary exposure might be underestimated due to the lack of comprehensive occurrence data for other foods. Significant data gaps exist for CPs occurrence in feed, their transfer to animal-derived food, and their effects on various species. As a result, EFSA could not establish health-based guidance values or conduct robust risk assessments for farm animals or companion animals. For SCCPs, a benchmark dose lower confidence limit (BMDL10) of 2.3 mg/kg body weight per day was identified based on the increased incidence of nephritis in male rats. Similarly, for MCCPs, a BMDL10 of 36 mg/kg body weight per day was established, reflecting the observed rise in relative kidney weights in both male and female rats. (EFSA, 2020).

### Accumulation properties of SCCPs and MCCPs

Understanding the differences in accumulation abilities between SCCPs and MCCPs is crucial for assessing their environmental and health risks. Both SCCPs and MCCPs are hydrophobic molecules, which is important for their industrial applications. CPs reduce friction and wear in machinery as additives in lubricants and metalworking fluids, and when used as flame retardants, their hydrophobicity helps form protective layers that inhibit ignition and slow fire spread (Nevondo and Okonkwo 2021). On the other hand, hydrophobicity allows CPs to interact with lipid-rich environments like biological membranes and organic matter. Consequently, CPs accumulate in these environments rather than staying in water (Shen et al. 2023). Their hydrophobic nature also affects their binding to proteins and other molecules (Gong et al. 2019).

The hydrophobicity of SCCPs and MCCPs differs as demonstrated by their different octanol–water partition coefficients (Kow). MCCPs have a higher log Kow (5.47–8.69) compared to SCCPs (4.39–8.69) (Hilger et al. 2011; Sijm and Sinnige 1995) which could lead to different accumulation abilities in tissues. Several studies have explored these differences in accumulation. Our findings align with previous research showing similar accumulation properties for both types of CPs. Mézière et al. (2021) found that SCCPs and MCCPs accumulate in similar concentrations in muscle, liver, adipose tissue, and serum of laying hens, indicating their passage through the gastrointestinal tract into the bloodstream and various tissues. Dong et al. (2020) studied the pharmacokinetics of SCCPs, MCCPs, and LCCPs in rats, showing peak concentrations in the liver, adipose tissue, blood, kidneys, and heart within 72 h, with the liver having the highest levels. CPs levels in all tissues plateaued after two weeks, and fecal excretion was identified as the primary elimination route. Du et al. (2020) observed in two snake species that SCCPs accumulated in the liver, while MCCPs with higher chlorination accumulated in muscle, and both MCCPs and LCCPs with higher chlorination levels accumulated in adipose tissue. Given the similar accumulation properties of MCCPs and SCCPs, it is crucial to consider extending restrictions to MCCPs to effectively address their potential environmental and health hazards.

### Accumulation of CPs in lipid-rich tissues

Our findings align with previous studies that show CPs primarily accumulate in lipid-rich tissues. Experiments with laying hens and broiler chickens revealed that SCCPs accumulate in proportion to the tissue’s lipid content. The highest accumulation was in adipose tissue, followed by the liver, yolk, and muscle, while leg meat, which has higher lipid content, showed more SCCPs than breast meat (Ueberschär and Matthes 2004; Ueberschär et al. 2007). In a study on Japanese quail, the distribution of ^14^C-CPs was tracked. Initially, radioactivity was high in metabolically active tissues like the liver, intestinal mucosa, spleen, bone marrow, and kidney. Over time, it increased in adipose tissue and other lipid-rich areas such as the uropygial gland. After 12 days, radioactivity decreased slowly even in adipose tissue, indicating it as a major storage site for CPs. The distribution patterns for SCCPs and medium-chain chlorinated paraffins (MCCPs) were almost identical (Biesssmann et al. 1982). Correlation of CPs levels with lipid content was also observed in fish (Sun et al. 2017) and black-spotted frogs (Du et al. 2019).

However, how CPs are distributed between different types of adipose tissue (AT) remains poorly understood. The two main types of AT are visceral adipose tissue (VAT) and subcutaneous adipose tissue (SAT). VAT, located adjacent to the intestine and connected to the liver through the portal vein, is metabolically active, secreting various adipokines that influence metabolism, inflammation, and insulin sensitivity. In contrast, SAT is less metabolically active, primarily functioning as an energy store and providing insulation (Lafontan 2013). Given VAT’s proximity to the intestine and the high affinity of CPs for lipids, it is plausible that CP-contaminated food would lead to rapid storage of these compounds in VAT after ingestion. This theory is supported by a Portuguese study showing that while levels of persistent organic pollutants (POPs) were positively correlated in both VAT and SAT, VAT exhibited significantly higher concentrations (Pestana et al. 2014). However, another study from Belgium, analyzing samples from obese individuals, found no significant differences in the concentrations of POPs, such as polychlorinated biphenyls (PCBs), dichlorodiphenyltrichloroethane, and polybrominated diphenyl ethers, between VAT and SAT (Malarvannan et al. 2013). Based on these findings, we hypothesize that VAT serves as the primary site for PCA accumulation, with SAT acting as a secondary, longer-term storage site. VAT's higher susceptibility to lipolysis could release stored POPs back into the bloodstream, and its greater blood flow and nerve supply may facilitate faster metabolic responses, potentially enhancing this process (Petrus et al. 2017).

Additionally, CPs may be transported in the bloodstream not only on albumin but also within lipoprotein particles such as chylomicrons, possibly leading to direct transport from the intestine to various tissues (Nauli and Matin 2019). In our study, the cultivation medium contained lipoproteins and about 3–5 g/l of albumin from fetal bovine serum, meaning there were more than 50 albumin molecules for each CPs molecule. Even with this albumin excess, CPs had ~ 600 times higher affinity for adipocytes than for the media, indicating that cellular lipid droplets are the primary storage sites for CPs in the body. Therefore, serum levels may not accurately reflect the total CPs content in the body but rather indicate recent exposures.

The long-term effects of CPs accumulation in lipid-rich tissues are not well understood and are difficult to predict. One potential issue is the prolonged retention of CPs, which may be linked to cancer (Bucher et al. 1987). During weight loss, persistent organic pollutants stored in adipose tissue can be released into the bloodstream, causing systemic toxicity. SCCPs have been reported to disrupt lipid membranes, increasing their permeability and alter integrity (Liu et al. 2021). The negative effects of CPs can be partly inferred from polychlorinated biphenyls (PCBs), which have similar properties and are also stored in adipose tissue. PCBs interfere with lipid metabolism, affecting their synthesis, uptake, and degradation, leading to disorders like obesity and chronic liver disease. PCBs also affect signaling in adipose tissue, contributing to insulin resistance, the development of diabetes, and impaired secretion of adipokines (Caron et al. 2020). Understanding these mechanisms is crucial for assessing the environmental and health risks associated with CPs exposure. This highlights the need for further research to fully grasp the implications of CPs accumulation in human tissues and the potential health risks involved.

### Chlorination effects on CPs accumulation and degradation

Our results reveal that chlorine content, not chain length, determines how CPS accumulate in adipocytes. Typically, accumulation studies measure the amount of a compound in cells after a set period. This measurement reflects two opposing processes: how the compound moves into the cells and accumulates, versus how it might be released back into the environment or broken down within the cells.

For CPs, chlorine content influences both accumulation and degradation processes. The hydrophobic nature of CPs, which increases with higher chlorine content is crucial for their ability to cross cell membranes. Hydrophobicity enhances CPs’ interaction with the lipid bilayer of cell membranes, affecting their diffusion and storage in lipid droplets (Huang et al. 2021). The exact mechanism why chlorination degree is important for the accumulation of CPs is not clear. It would be possibly interplay of multiple factors. However, we know that higher degrees of chlorination increase the lipophilicity of CPs molecules (Liu et al 1994). Lipophilic compounds preferentially partition into lipid-rich environments, such as cell membranes and intracellular lipid droplets (Liu et al. 2011). This can lead to higher accumulation of chlorinated compounds in cells with significant lipid content, such as adipocytes.

Chlorination also affects CPs degradation. Studies show that SCCPs with lower chlorine content are broken down to CO_2_ and thus appear at lower concentrations in tissues compared to those with higher chlorine content or MCCPs (Darnerud et al. 1982). When SCCPs, MCCPs, and LCCPs with varying chlorination levels were incubated with human liver microsomes, their carbon chains were shortened. It has been suggested that chlorine atoms play a key role in their breakdown and cytochromes were identified as crucial enzymes in CPs degradation (He et al. 2021). Reduced elimination rates based on carbon chain length have also been observed in chicken liver (Huang et al. 2021). Although some degradation of CPs likely occurs in adipocytes, because these cells contain cytochrome P450 enzymes, such as CYP1A1 and CYP2U1 (Ellero et al. 2010). However, the extent of this degradation is still unclear. Thus, the higher accumulation of CPs with more chlorine atoms can be attributed to their enhanced transport and storage due to increased hydrophobicity, along with potentially reduced degradation rates.

### Limitations of the study

While this study provides valuable insights into the accumulation of CPs, a significant environmental contaminants in mammalian fat cells, several limitations should be acknowledged. The use of an in vitro system, while useful for isolating cellular mechanisms, may not fully replicate the complex interactions present in a whole organism. The concentrations of CPs used in this study were selected to assess the potential for accumulation; however, these levels may not reflect typical exposure scenarios. The time frame for contaminant exposure in vitro was limited to 1 week, which may not represent longer-term accumulation and its associated effects. While our findings indicate significant accumulation of PCA, this study does not delve into the specific mechanisms of uptake within adipocytes.

## Conclusion

Our study reveals the accumulation potential of SCCPs and MCCPs in an in vitro model of 3T3-L1 cells which could be used in the process of consideration of MCCPs restriction, because the extensive accumulation of CPs might lead to chronic toxicity over time.

Interestingly, MCCPs originally chosen as an alternative to SCCPs, show a similar tendency to accumulate in 3T3-L1 cells as SCCPs. This accumulation correlates with the intracellular lipid content, favoring the entry of SCCPs and MCCPs into adipocytes over preadipocytes. Based on GC–NCI–HRMS analysis of CPs congeners accumulated intracellularly over time and linear regression modeling of their relative quantities, we demonstrate the significant role of chlorination, but not chain length, in the cellular accumulation of CPs.

## Supplementary Information

Below is the link to the electronic supplementary material.

Supplementary file1 Suppl. Fig. 1. Relative loss of CPs from wells in the absence of cells. 2 ml of cultivation medium with different concentrations of CPs was incubated in 37 °C for 24 h. After this period, volume of medium was measured and CPs in this volume were quantified. Data are presented as mean ± SD, *n* = 2. (JPG 90 KB)

Supplementary file2 Suppl. Fig. 2. Amount of triacylglycerols in 3T3-L1 cells. Preadipocytes or differentiated adipocytes were used and CPs were added to the cultivation media at a concentrations of 500 ng/ml. Media were exchanged every 12 h, and cells were harvested after 1, 2, 3, 4, 5, or 6 days after the first addition of CPs. The amounts of triacylglycerols measured using enzymatic kit (Erba Lachema). Data are presented as mean ± SD, *n* = 2. (JPG 102 KB)

## Data Availability

The datasets generated and analysed during the current study are available from the corresponding author on reasonable request.

## Abbreviations

CS: Calf serum
CPs: Chlorinated paraffins
Dex: Dexamethasone
DMI: Differentiation medium I
DMII: Differentiation medium II
FBS: Fetal bovine serum
IBMX: 3-Isobutyl-1-methylxanthine
Ins: Insulin
MCCPs: Medium-chain chlorinated paraffins
SCCPs: Short-chain chlorinated paraffins
LCCPs: Long-chain chlorinated paraffins
PBS: Phosphate-buffered saline
PCBs: Polychlorinated biphenyls
