# Supplementary figures and images for: Accumulation of chlorinated paraffins in adipocytes is determined by cellular lipid content and chlorination level
Source: Arch Toxicol. 2025 Jan 10;99(3):1117–31. doi: 10.1007/s00204-024-03956-3 (PMC11821709; doi:10.1007/s00204-024-03956-3)

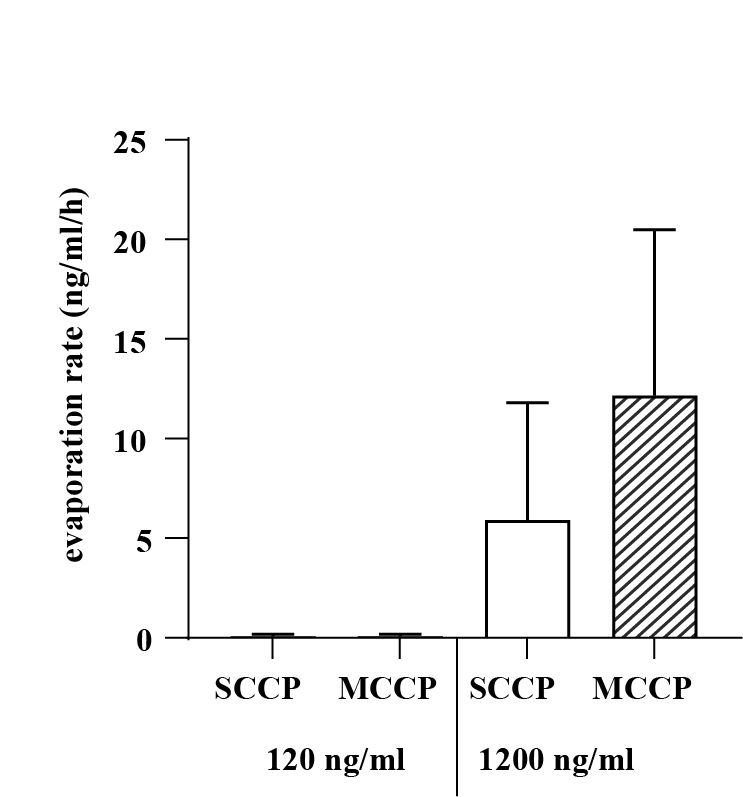

Supplement: Supplementary file 1 — Supplementary file1 Suppl. Fig. 1. Relative loss of CPs from wells in the absence of cells. 2 ml of cultivation medium with different concentrations of CPs was incubated in 37 °C for 24 h. After this period, volume of medium was measured and CPs in this volume were quantified. Data are presented as mean ± SD, n = 2. (JPG 90 KB) [file 204_2024_3956_MOESM1_ESM.jpg]

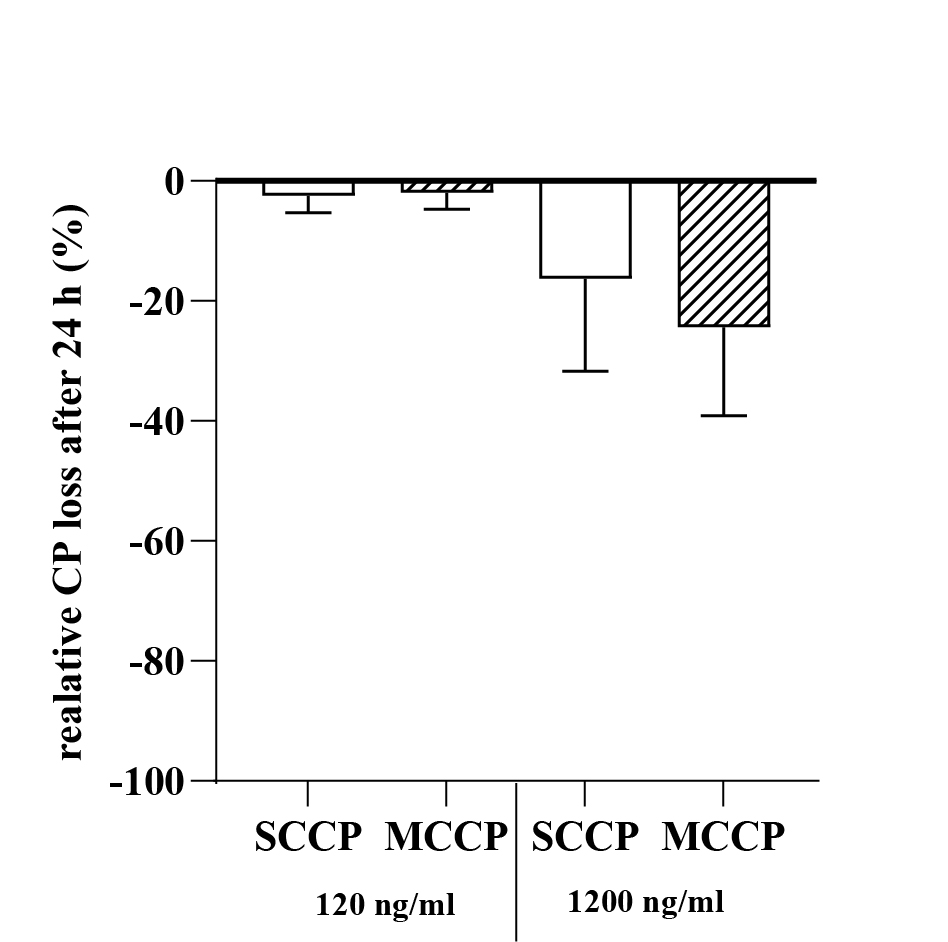

Supplement: Supplementary file 2 — Supplementary file2 Suppl. Fig. 2. Amount of triacylglycerols in 3T3-L1 cells. Preadipocytes or differentiated adipocytes were used and CPs were added to the cultivation media at a concentrations of 500 ng/ml. Media were exchanged every 12 h, and cells were harvested after 1, 2, 3, 4, 5, or 6 days after the first addition of CPs. The amounts of triacylglycerols measured using enzymatic kit (Erba Lachema). Data are presented as mean ± SD, n = 2. (JPG 102 KB) [file 204_2024_3956_MOESM2_ESM.jpg]
